# Biodegradation, Angiogenesis, and Inflammatory Response of a Collagen-Chitosan-Polyvinyl Alcohol (PVA) Membrane: *In Vivo* Model of Guided Tissue Regeneration

**DOI:** 10.1055/s-0044-1801305

**Published:** 2025-04-16

**Authors:** Ira Komara, Furi Andanawari, Agus Susanto, Euis Reni Yuslianti, Ina Hendiani, Prajna Metta, Amaliya Amaliya

**Affiliations:** 1 Department of Periodontics, Faculty of Dentistry, Universitas Padjadjaran, Bandung, Indonesia; 2 Post-Graduate Program in Periodontology, Dental Faculty, Universitas Padjadjaran, Bandung, Indonesia; 3 Department of Oral Biology and Biomedical, Faculty of Dentistry, Universitas Jenderal Achmad Yani, Cimahi, Indonesia

**Keywords:** angiogenesis, biodegradation, chitosan, collagen, guided tissue regeneration, inflammatory response

## Abstract

**Objective:**

The aim of this study was to examine the biodegradation, angiogenesis, and inflammatory response in collagen-chitosan-polyvinyl alcohol (PVA) membranes.

**Materials and Methods:**

This study employed an experimental approach utilizing a randomized controlled trial design. Wistar rats were used as subjects, with 51 rats divided into three groups. Each group received a different treatment: application of the collagen-chitosan-PVA membrane, pericardial membrane, or cross-linked pericardial membrane, administered subcutaneously. On days 0, 7, 14, and 30, the rats were terminated, and the membranes and surrounding tissues were collected for analysis. A histological examination was performed to evaluate the membrane biodegradation rate, the number of blood vessels formed, and the inflammatory response.

**Statistical Analysis:**

The data were analyzed using the Kruskal-Wallis and Mann-Whitney tests, with a *p* -value of < 0.05 considered statistically significant.

**Results:**

The collagen-chitosan-PVA membrane remained in the tissue up to day 30, while the pericardial membrane and cross-linked pericardial membrane were completely degraded by day 7. The average number of new blood vessels formed in the collagen-chitosan-PVA membrane on days 7, 14, and 30 was greater than that in the pericardial membrane and cross-linked pericardial membrane, which was statistically significant ( *p* < 0.005). The average number of inflammatory cells in the collagen-chitosan-PVA membrane on day 30 was lower than that in the pericardial membrane and cross-linked pericardial membrane, which was statistically significant ( *p* < 0.005) for neutrophils, monocytes, and lymphocytes. However, the difference was not statistically significant ( *p* > 0.05) for eosinophils and mast cells.

**Conclusion:**

Biodegradation, angiogenesis, and the inflammatory response in collagen-chitosan-PVA membranes showed better results compared with other membranes. Collagen-chitosan-PVA membranes exhibit potential for application in guided tissue regeneration treatment for periodontal disease.

## Introduction

A barrier membrane is commonly used in periodontal regenerative therapy, such as guided tissue regeneration (GTR). The placement of membranes aims to prevent epithelial growth toward the apical direction so that the space during cementum formation, periodontal tissue adhesion, and new bone formation in the teeth suffering from defects can be maintained. 1 2 The membrane selection by considering its biological and mechanical characteristics is important to obtain the optimal treatment results. The characteristic membranes must be biocompatible, nontoxic to the patient's body, have adequate mechanical and physical properties, have appropriate degradation time for tissue formation, have cell occlusivity, be able to facilitate the proliferation of regenerative cells, promote angiogenesis, and vascularize tissue regeneration. 3

GTR membranes are classified according to their degradation properties, specifically as resorbable and nonresorbable. Resorbable membranes are preferred since they eliminate the need for a secondary surgery for removal. Collagen is a natural material often used in the making of resorbable membranes since it has good biological activities, such as hemostasis, biocompatibility, and biodegradability, but it degrades quickly before the periodontal tissue completely regenerates. 4 Various collagen membranes found in the market include pericardium membrane and cross-linked pericardium membrane.

The pericardial membrane (PM) is a resorbable membrane derived from cow and pig pericardium containing collagen and has been widely used in periodontal tissue regenerative therapy. The PM shows a longer and more stable resorption pattern because it comes from a compact area so its physical and mechanical properties are better, but it shows slow vascular penetration that will hinder angiogenesis. 5 The degradation time of the collagen membranes can be prolonged through cross-linking methods using physical or chemical procedures, such as ultraviolet light, *hexamethylene diisocyanate* , glutaraldehyde plus irradiation, and *diphenylphosphoryl azide* , but the process can reduce the tissue integrity, decrease vascularization and biocompatibility, and increase the inflammatory response. 5 6 Based on these considerations, in this study the collagen membrane is integrated with polymer, which is chitosan and polyvinyl alcohol (PVA) expected to be able to meet the membrane characteristics required in regenerative therapy.

Chitosan in dental medicine is used as a bone regeneration material and to improve the properties of other dental materials. Chitosan is a natural polymer of the polysaccharide type deriving from crustacean exoskeletons, such as shrimps, crabs, or shellfish. As a cross-linked agent, chitosan shows good biocompatibility, biodegradability, and antimicrobial activities. 7 8 Besides that, chitosan is proven to have a pore structure that can facilitate differentiation and cell proliferation as well as neovascularization. 9 However, the weakness of chitosan is its vulnerability, so it is necessary to choose an additive that is compatible with a high mechanic characteristic, such as PVA. 10 11 12 PVA is a water-soluble synthetic polymer with very good biocompatibility, can be easily processed, and is biodegradable with superior mechanical properties, so it can be used as a blend to enhance the mechanical characteristics. 13 14

The collagen mixture with polymer has the potential to be developed as the alternative membrane for GTR. In a previous study, physical and mechanical properties were evaluated in the collagen-chitosan-PVA membrane (CM), which had been irradiated with gamma rays. The results indicated that gamma-ray irradiation had a positive effect on enhancing the mechanical properties of the membrane. Additionally, scanning electron microscopy morphological analysis revealed the presence of microstructural pores, which could facilitate vascularization. 10

No studies have been conducted on CMs associated with GTR, prompting an *in vivo* investigation on Wistar rats to investigate their impact on periodontal tissue regeneration. This study aims to examine biodegradation, angiogenesis, and the inflammatory response in CMs.

## Materials and Methods

This study is an experimental study with a randomized control trial design using animals as the subjects, which are 51 Wistar rats calculated with the Federer formula previously. The rats selected were Wistar male white rats, with a weight of 150 to 250 g, aged 6 to 8 weeks, and in good health condition marked with active movement, responding well when stimulated, no anatomy disorder, and no hair loss. Rats experiencing weight loss, illness, or death during acclimatization were not involved in this study.

This study has obtained ethical approval from the Health Research Ethical Committee of the Faculty of Medicine of Universitas Jenderal Achmad Yani (Unjani) Cimahi with the number 001/UH4.08/2022. The use of animals as the subjects of the research considers three main principles regarding animal use, which are to substitute humans with rats as the subjects of the research because they have almost similar physiological conditions with humans (replacement), to determine the limited quantity of animals used (reduction), and to treat the animals used properly or ethically to meet the concept of animal testing, which is to avoid pain in them (refinement).

Fifty-one Wistar rats were randomly divided into three test groups and two control groups. In the test groups, Wistar rats were treated by placing CM, PM, and cross-linked pericardial membrane (CLPM). The negative control (NC) group of Wistar rats only went through surgery without placing the membranes, while the normal control group did not receive any treatment. Subsequently, these Wistar rats were terminated on days 0, 7, 14, and 30 to assess the level of biodegradation, the quantity of newly formed blood vessels, and the inflammatory response.

## Surgery Procedure

The experimental animals involved in this study had gone through the adaptation time for 1 week at the Laboratory of Animal Experiments of the Faculty of Medicine of Unjani. Those animals then went through rescreening 1 day before and 1 day after the treatment. Prior to the procedure, the rats were cleaned, and they were anesthetized using ketamine hydrochloride with a dose of 45 mg/kg of body weight and xylazine as much as 0.35 mg/kg of body weight intramuscularly at the abdominal muscles. The incision was made at the dorsum as long as 10 mm up to the subcutaneous layer under the dermis, and then for the test group the CM membrane, PM, and CLPM were placed, followed by simple interrupted suturing of the incision area.

Termination was performed by putting the rats into a chamber with CO _2_ inhalation, and then the membranes and tissue surrounding them were taken. The CM group, PM group, CLPM group, and NC group were terminated on days 0, 7, 14, and 30, while the normal control group (N) was terminated on day 0.

### Membrane Biodegradation Analysis

Membrane biodegradation is the process of lacking the membrane thickness due to the enzymatic activity. The application area on the rat's dorsum was cut transversally with the size of 12×12×5 mm and taken, and then the remaining of membrane thickness was measured using the Image J computer program on days 0, 7, 14, and 30.

### Histological Analysis

The membranes and surrounding tissue were fixed in 10% neutral-buffered formalin solution for 24 hours, and then we used the solution consisting of alcohol 70%, alcohol 80%, alcohol 90%, alcohol anhydrides, toluene, and paraffin wax for dehydration and classification, gradually over 1 day. The organ sample was sealed with an embedded device filled in liquid paraffin and refrigerated. A microtome with a thickness of 5 *μ* m was used to slice the cold block. These sections were stained with hematoxylin and eosin for histopathological analysis..

The number of new blood vessels formed was calculated manually under a binocular microscope at 400× magnification from 5 fields of view of slide preparation. The inflammatory response was observed through the calculation of the spread of the inflammatory cells, which are eosinophil, monocyte, neutrophil, lymphocyte, and mast cells under the binocular microscope at 100× magnification from 5 fields of view of slide preparation.

## Statistical Analysis

In this study, the data obtained consisted of quantitative measurements. The normality of the data was tested using the Kolmogorov–Smirnov test, which indicated that the data were not normally distributed. Therefore, the analysis was performed using a nonparametric analysis of variance, specifically the Kruskal–Wallis test. Subsequently, post hoc analysis using the Mann–Whitney test was conducted to determine which groups differed significantly at each observation time. The significance of the results was assessed based on a *p* -value of < 0.05.

## Results

**Fig. 1:**
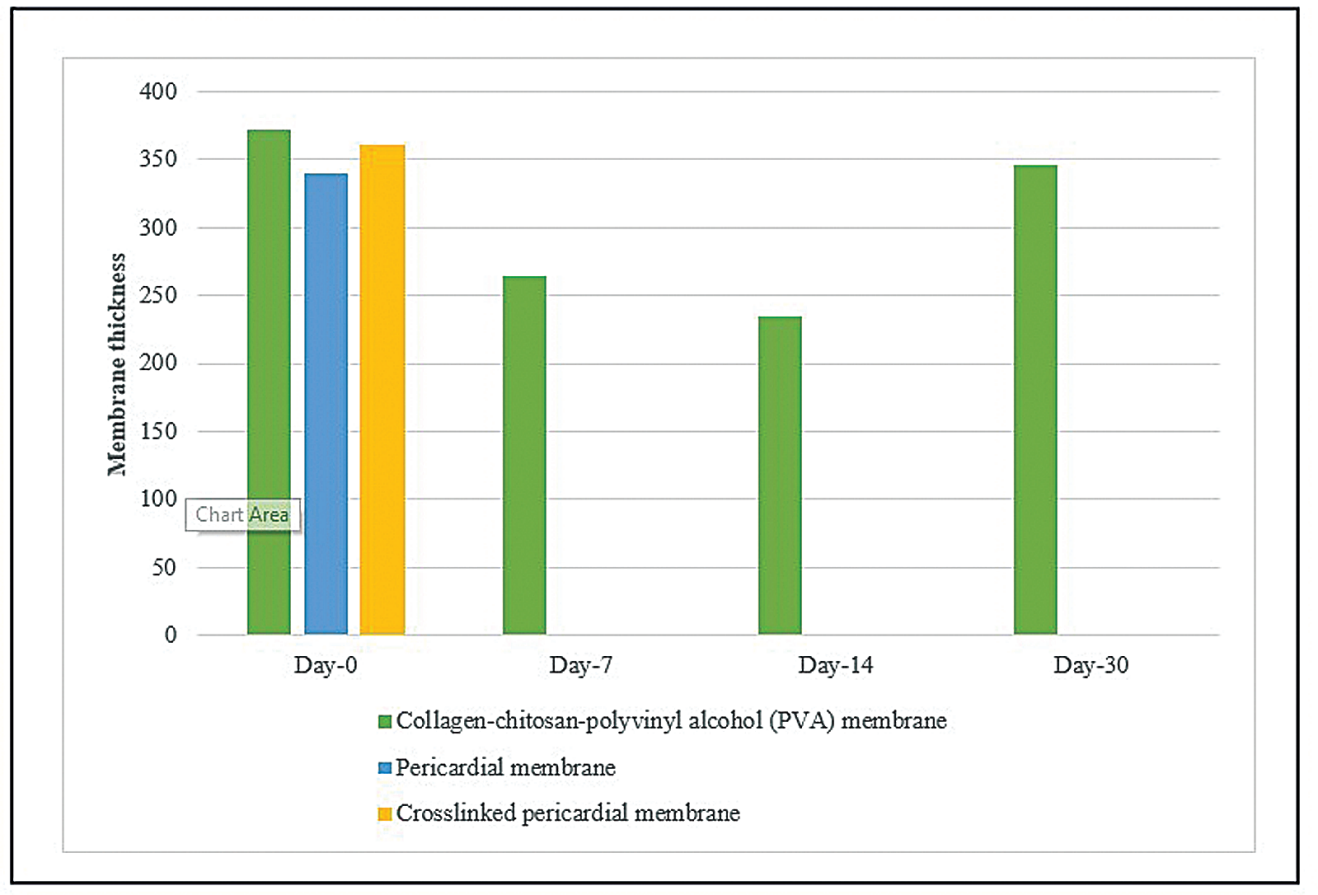
The mean value of membrane thickness for Collagen-chitosan-polyvinyl alcohol membrane (CM), Pericardial membrane (PM), and Crosslinked pericardial membrane (CLPM) on days 0, 7, 14, and 30.

The membrane biodegradation rate was calculated by measuring the remaining membrane thickness. The measurement results in the CM, PM, and CLPM on days 0, 7, 14, and 30 are presented in Fig. 1 . On day 7, the PM and CLPM groups were degraded completely, while the CM group remained intact until day 30.

**Fig. 2:**
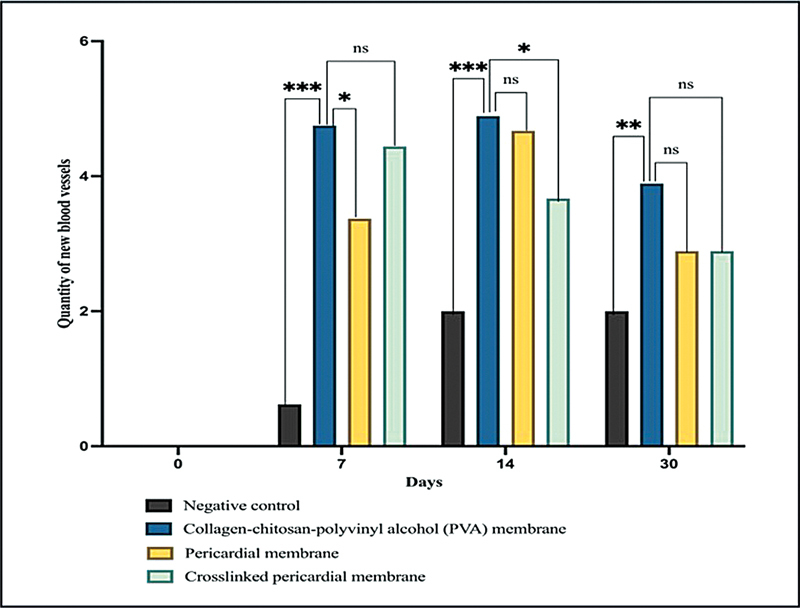
The mean value of new blood vessels in the negative control (NC), collagen-chitosan-PVA (CM), pericardial (PM), and cross-linked pericardial (CLPM) membrane groups (***: *p* < 0.001; **: *p* < 0.01; *: *p* < 0.05; ns: insignificant difference).

Angiogenesis began on the 7th day in both the experimental and control groups. The average number of the quantity of new blood vessels formed in the CM group was higher compared with the PM group, CLPM group, and NC group in all the time of the observation, where it shows that there is a significant difference statistically, with *p* -value < 0.05, as presented in Fig. 2 .

Based on the post hoc test, on day 7, there was a significant difference between the CM group and NC group ( *p* = 0.001) and the PM group ( *p* = 0.027), but there was no significant difference between the CM group and CLPM group ( *p* = 0.546). On day 14, there was a significant difference between the CM group and NC group ( *p* = 0.001) and the CLPM group ( *p* = 0.024), but there was no significant difference between the CM group and PM group ( *p* = 0.609). On day 30, there was a significant difference between the CM group and NC group ( *p* = 0.004), but there was no significant difference between the CM group and PM group ( *p* = 0.168) or CLPM ( *p* = 0.110).

**Fig. 3:**
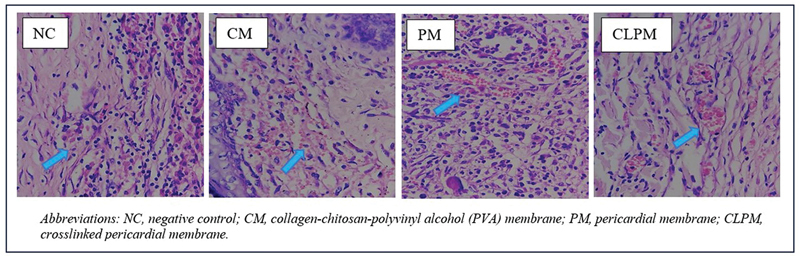
The histological picture in the NC, CM, PM, and CLPM groups on the 7th day with the coloring of Hematoxylin Eosin with the enlargement of 400X; the blue arrow mark shows new blood vessels.

In the histological image, it can be observed that the distribution of new blood vessels in the CM group is greater compared with the NC, PM, and CLPM groups, as shown in Fig. 3 .

**Fig. 4:**
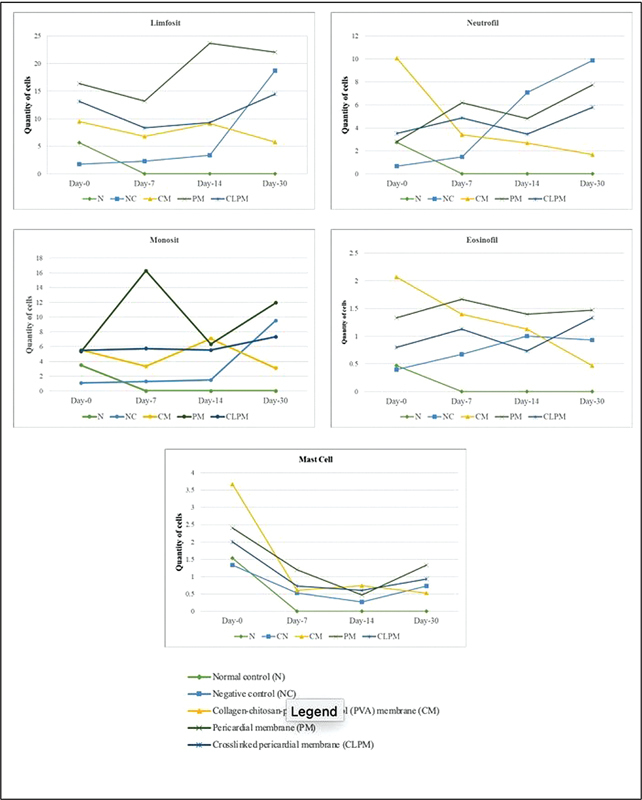
The mean value of inflammatory cells in the normal control group (N), negative control (NC), collagen-chitosan-polyvinyl alcohol membrane (CM), pericardial membrane (PM), and crosslinked pericardial membrane (CLPM) on days 0, 7, 14, and 30).

The inflammation response indicates how the tissue reacts to the membrane, as observed through eosinophils, neutrophils, monocytes, lymphocytes, and mast cells. Initially, on day 0, the CM group had the highest average number of inflammatory cells compared with the N, NC, PM, and CLPM groups, and it was significant statistically ( *p* = 0.000). Along with the time progress of the observation, in the CM group the quantity of inflammatory cells tended to decrease compared with the NC, PM, and CLPM groups, which experienced an increase, and this can be seen in Fig. 4 . This pattern was further evident in the average quantity on day 30, where the CM group had fewer inflammatory cells compared with the NC, PM, and CLPM groups, and it was significant statistically in the eosinophil cell ( *p* = 0.005), neutrophil cell ( *p* = 0.000), lymphocyte cell ( *p* = 0.000), and monocyte cell ( *p* = 0.000), but it was not significant statistically in the mast cell ( *p* = 0.058).

**Fig. 5:**
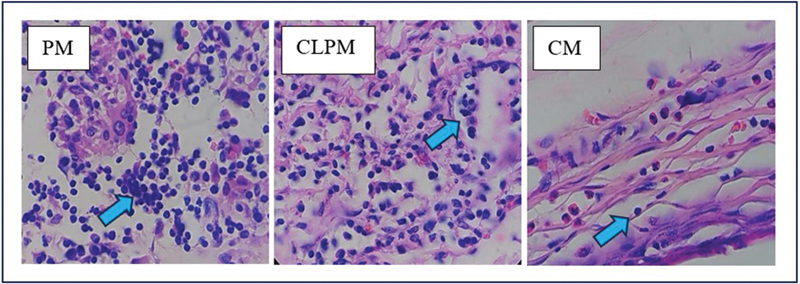
The histological picture in the CM, PM, and CLPM groups on the 30 th day with the coloring of Hematoxylin Eosin with the enlargement of 100X; the blue arrow mark shows the inflammatory cells.

In the histological picture on the 30th day, the amount of the inflammatory cell spread in the CM group was less than that in the NC, PM, and CLPM groups, and it can be seen in Fig. 5 .

## Discussion

This study compares the biodegradation rate, quantity of new blood vessels, and inflammatory response through the observation of inflammatory cell spread between the CM, the PM, and the CLPM inserted into the experimental Wistar rats. The ideal membrane characteristics that are required to support the success of GTR, include the ability to maintain space during the periodontal tissue regeneration process, facilitate the proliferation of regenerative cells, enhance angiogenesis and vascularization for tissue regeneration, ensure good biocompatibility, and possess adequate physical and mechanical properties. 3 4 15

The findings regarding membrane biodegradation rates indicate that the CM degrades more slowly than both the PM and the CLPM. The addition of PVA to the CM can enhance its mechanical properties by improving tensile strength and elongation at break, which influences the degradation process. 10 Our results are in line with a previous study from Zhou et al 16 that added PVA to the collagen-PVA membrane to slow down the degradation time. Zhuang et al 17 also reported that there is an increase in tensile strength in a wet condition by adding PVA to the chitosan membrane. Tensile strength and elongation at break indicate that the strength and elasticity of a membrane become important physical parameters in supporting its application. 18 The biodegradation rate of the GTR membrane has to be able to be controlled at least 4 to 6 weeks along with the healing rate to achieve the optimal periodontal tissue regeneration. 15 19 20 This is consistent with research findings indicating that the CM remains intact until week 4.

Ideal GTR membranes, besides having an appropriate degradation rate for periodontal tissue growth, also stimulate the differentiation and proliferation of tissue regeneration cells, including new blood vessels that supply oxygen and nutrients to the growing tissue. In the wound-healing process, angiogenesis begins on day 7, where endothelial cells migrate to the extracellular matrix area temporarily in the wound area, which will then form new blood vessels. 21 22 23 The study results show that new blood vessels were already formed on the 7th day in all groups with the average quantity of new blood vessels in the CM group being much more than that in the PM and the CLPM groups. Rothamel et al, 24 in their study on the cow PM, reported that pericardial tissue has good physical and mechanical properties, but it shows slow vascular penetration. Previously, Rothamel and colleagues 24 reported that there is retardation of the angiogenesis process in the pericardial collagen membrane because the PM structure is thicker without any pores, while the collagen membrane structure has more pores. Based on that, the angiogenesis pattern can be influenced by the membrane structure.

The PM originates from a compact area, resulting in slow vascular penetration which impedes angiogenesis. 5 Porosity in the membrane allows for the infiltration of nutrients into the defect area, which can support tissue regeneration. Chitosan can be produced in a porous structure enabling to do cell proliferation, cell migration, nutrient exchange, and angiogenesis. Based on the previous study, the CM shows a microarchitecture of pore, with pores sizes ranging from 1 to 50 µm, and pore interconnectivity, potentially providing space for vascularization. 3

Another GTR membrane characteristic that must be owned is good biocompatibility to enable integration with host tissue without causing an inflammatory response or immunity response to appear, which can disturb healing and cause harm to patients. 15 25 The inflammatory cells involved in wound healing are primarily polymorphonuclear leukocytes (PMNs). Leukocytes can be found in the inflammation, proliferation, and remodeling phases. During the inflammation phase, leukocytes migrate to the extravascular area guided by chemotactic mediators released by damaged tissues. In this phase, PMNs play a significant role in the phagocytosis process. Neutrophil is an inflammatory cell appearing for the first time after injury, and it has the ability to produce mediators that kill bacteria. Neutrophils are particularly prominent in the inflammatory and early proliferation phases. 26 27 28 PMNs that are seen up to the end of the proliferation phase and the remodeling phase can be the indicator of the persistent inflammation reaction, and if this is allowed to continue, it will become chronic inflammation; meanwhile, the macrophage is discovered in all three phases of wound healing and plays a greater role in the proliferation and the remodeling phase. If leukocytes are still abundant at the end of the proliferation and the remodeling phase, it is possible that the inflammatory process is still ongoing and tissue damage is still occurring. 29 30

Based on the data, in the CM group the quantity of cells decreases over time, while in other groups, there are fluctuations of increase and decrease. Therefore, it can be concluded that the wound-healing process in the CM group is relatively faster compared with the PM and CLPM groups. This is in line with the study of Kusumastuti et al 31 who stated that the number of inflammatory cells will keep decreasing along with the time because the wound-healing process is taking place. This wound-healing process will be hindered if inflammatory cells keep increasing. Wahyuningsih 32 reported that the mast cell given in the castor bean plant extract experienced a decrease in the number of cells faster. This shows an indication that the wound-healing process is faster.

The chitosan content in the CM has a strong anti-inflammatory activity, which can inhibit the growth of bacteria and fungi. 32 The study by Hartatiek et al 33 showed that there is an antibacterial activity decrease of *Escherichia coli* and *Staphylococcus* *aureus* in the nanofiber composite of collagen-chitosan-PVA along with the chitosan concentration decrease. Zheng et al 34 reported that the anti-inflammatory activity occurs in chitosan because of its polycationic trait and the impermeable membrane is formed due to the interaction between the cationic group in chitosan and the anionic group on the cell surface that can hinder nutrition transport. Chitosan with low molecule weight can enter the bacterial cells, destroying microbe cells and disturbing the metabolism.

In addition to its antibacterial effects, chitosan also has an antiallergic effect by inhibiting histamine release. Eosinophil and mast cells play a role in the allergic process. The granule in the mast cell contains histamine, when reacting with an antigen, the mast cell releases its granule content indicating that the increase of the mast and eosinophil cell quantity shows that there is an allergic reaction. 29 In this study, the quantity of eosinophil and mast cells in the CM group decreased over time, while in the PM and CLPM groups, it increased. This indicates that there was no allergic reaction in the collagen-chitosan-PVA.

The CM is expected to serve as an alternative membrane in the GTR process. However, in this study, it was limited only to soft tissue without involving the hard tissue, therefore, further study is necessary to investigate bone defects in experimental animals to discover its influence toward the alveolar bone remodeling process.

## Conclusion

Biodegradation, angiogenesis, and the inflammatory response in CMs showed better results compared with other membranes. CMs exhibit potential for application in GTR treatment for periodontal disease.
